# Cost-effectiveness of adding empagliflozin to the standard therapy for Heart Failure with Preserved Ejection Fraction from the perspective of healthcare systems in China

**DOI:** 10.3389/fcvm.2022.946399

**Published:** 2022-09-02

**Authors:** Yaohui Jiang, Jun Xie

**Affiliations:** Department of Cardiology, Nanjing Drum Tower Hospital, Affiliated Hospital of Medical School, Nanjing University, Nanjing, China

**Keywords:** empagliflozin, Heart Failure with Preserved Ejection Fraction, China, cost-effectiveness analysis, healthcare systems

## Abstract

**Background:**

The Empagliflozin Outcome Trial in Patients with Chronic Heart Failure with Preserved Ejection Fraction (EMPEROR-Preserved) is the first randomized controlled trial to provide promising evidence on the efficacy of adding empagliflozin to the standard therapy in patients with Heart Failure with Preserved Ejection Fraction (HFpEF), but the cost-effectiveness of add-on empagliflozin treatment remains unclear.

**Method:**

A Markov model using data from the EMPEROR-Preserved trial and national database was constructed to assess lifetime costs and utility from a China healthcare system perspective. The time horizon was 10 years and a 5% discount rate was applied. Incremental cost-effectiveness ratio (ICER) against willingness to pay (WTP) threshold was performed to evaluate the cost-effectiveness. A series of sensitivity analyses was applied to ensure the robustness of the results.

**Results:**

Compared to standard therapy, the increased cost of adding empagliflozin from $4,645.23 to $5,916.50 was associated with a quality-adjusted life years (QALYs) gain from 4.70 to 4.81, projecting an ICER of $11,292.06, which was lower than a WTP threshold of $12,652.5. Univariate sensitivity analysis revealed that the parameters with the largest impact on ICER were cardiovascular mortality in both groups, followed by the cost of empagliflozin and the cost of hospitalization for heart failure. Probabilistic sensitivity analysis indicated that when the WTP threshold was $12,652.5 and $37,957.5, the probability of being cost-effective for adding empagliflozin was 52.7% and 67.6%, respectively. Scenario analysis demonstrated that the cost of empagliflozin, the cost of hospitalization for heart failure, NYHA functional classes, and time horizon had a greater impact on the ICER.

**Conclusion:**

At a WTP threshold of $12,652.5, the add-on empagliflozin treatment for HFpEF was cost-effective in healthcare systems in China, which promoted the rational use of empagliflozin for HFpEF.

## Introduction

Heart failure (HF) has become a growing global public health problem which is a severe clinical manifestation and/or end stage of various cardiac diseases and is a leading cause of hospitalization and death (1). HF is divided into heart failure with mildly reduced ejection fraction (HFmrEF), Heart Failure with Preserved Ejection Fraction (HFpEF), and heart failure with reduced ejection fraction (HFrEF). The clinical characteristics of HFmrEF are more similar to HFpEF. The rate of HF in the United States and Europe ranges from 1 to 14%, of which nearly half are HFpEF (2, 3). The prevalence of HF among adults >35 years old in China is about 1.3%, of which 0.3% is HFpEF (3). The associated HF global economic burden is estimated at $108 billion every year with the most costs of HF deriving from hospitalizations (4). With the aging population in China and the increase in risk factors such as hypertension and coronary heart disease (CHD), the prevalence of HFpEF is on the rise, and the direct and indirect costs of HFpEF will increase significantly in the future.

We have been trying to find a treatment strategy for HFpEF, but compared with the breakthroughs in the treatment of HFrEF, the CHARM-Preserved trial, the TOPCAT trial, and the PARAGON-HF trial showed that candesartan, spironolactone, and SAC/VAL did not reduce the risk of CV or hospitalization for HF among patients with HFpEF which were not satisfactory results (5–7). This reflected the different pathophysiological processes between HFpEF and HFrEF, the mechanism except for the activation of renin-angiotensin aldosterone system (RASS) has not been explored and new therapeutic targets have not been found. Sodium-glucose cotransporter 2 (SGLT-2) has been used as a novel therapy for type 2 diabetes mellitus (T2DM), inhibiting the proximal renal tubular SGLT protein family reabsorption of glucose (8). The Empagliflozin Outcome Trial in Patients with Chronic Heart Failure with Preserved Ejection Fraction (EMPEROR-Preserved) could reduce the risk of cardiovascular (CV) death or hospitalization for HF in patients with HFpEF (9). Some animal experiments found that there were benefits of HF independently of blood glucose reduction, including higher circulating ketone levels, natriuresis, anti-inflammatory effects, and inhibiting RASS (10, 11). It was noteworthy that the United States Food and Drug Administration (FDA) has approved that empagliflozin could be treated for HFpEF.

However, guidelines emphasized sacubitril/valsartan (SAC/VAL), angiotensin-converting enzyme inhibitors (ACEI), angiotensin receptor blockers (ARB), beta-blockers, and spironolactone for comorbidities for HFpEF (12), and the cost of HFpEF will increase significantly. Previous cost-effective studies focused on HFrEF or HF as a homogeneous group, lacking cost-effective evaluations for HFpEF, financial burden and life quality of HFpEF differed from HFrEF, and higher comorbidity in HFpEF contributed to higher CV-related costs (13). To fill the gap, we are the first to evaluate the cost-effectiveness of the add-on empagliflozin treatment for HFpEF, our study aims to help clinicians and decision-makers judge the economic value of this new therapy.

## Method

### Model structure

**Figure 1:**
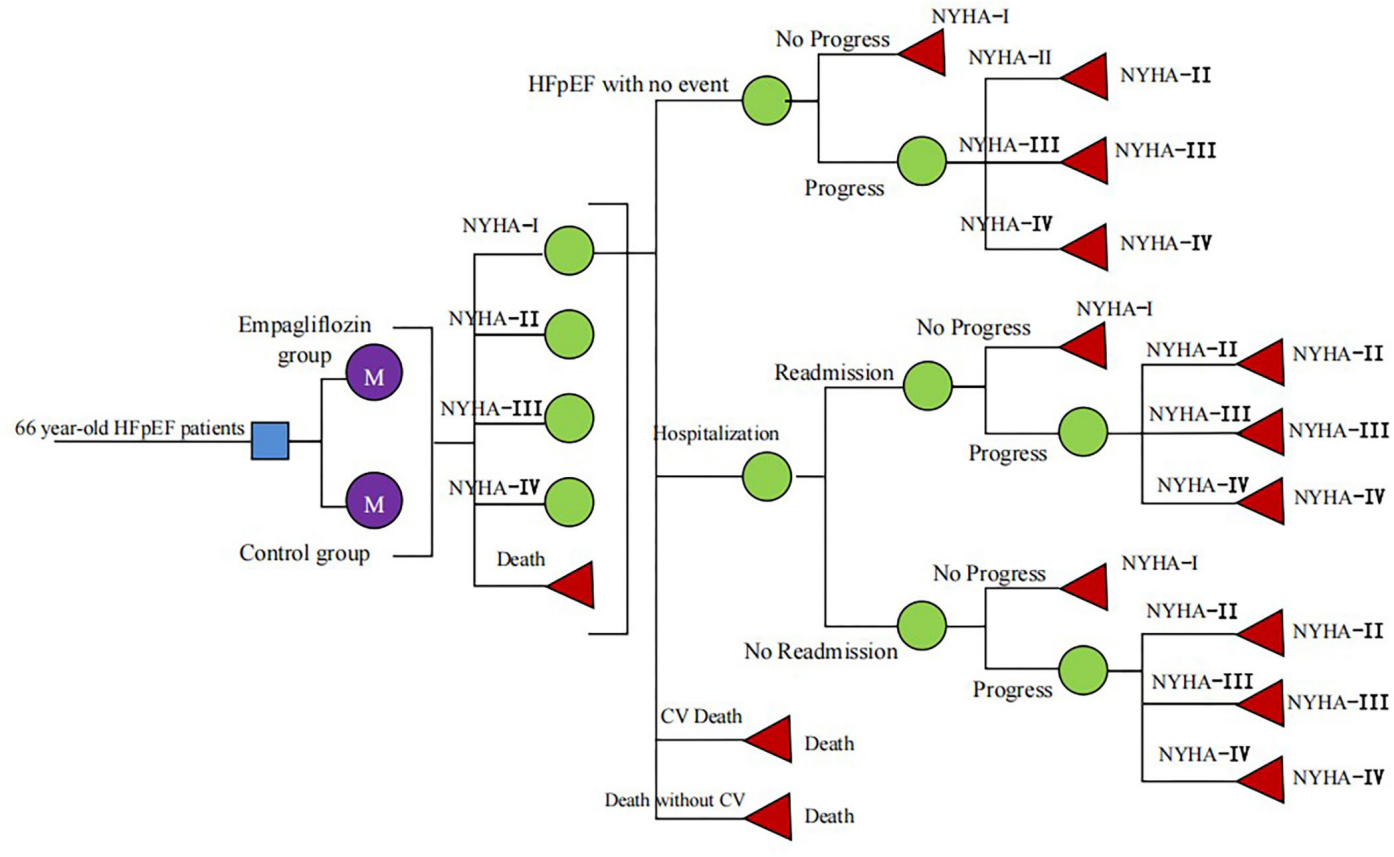
Schematic representation of the Markov model.

A Markov model was constructed to evaluate the cost-utility of different therapies for HFpEF: standard therapy and standard therapy combined with empagliflozin (10 mg, once daily). According to the history of HFpEF, HFpEF was divided into five states: New York Heart Association (NYHA) function class I, II, III, and IV and death (Figure 1), in which the death was absorption state (14). The patients would be in the same New York Heart Association (NYHA) function classes or they would change the NYHA function classes at the end of each cycle, indicating that the symptom would be improved or worsened. Despite standard HF therapy, the rate of readmission for HF within 3 months of discharge for hospitalized patients approached 30% (15), and we hypothesized that all readmissions related to HF occurred within 3 months in patients who had experienced high-frequency hospitalization. Clinical outcomes included hospitalization for HF, readmission for HF, CV death, and non-CV death. The model was conducted by Excel 2016 software. The simulation time was 10 years and the cycle was set as 3 months (90 days), and the half-cycle correction was applied to prevent overestimation of the expected survival time. A 5% discount rate was also applied according to the recommendations of the Chinese Pharmacoeconomic Evaluation Guide 2019 (16) (Table 1).

**Table 1:** Selected model inputs.

**Variables**	**Value**	**Range**	**Distribution**	**References**
Clinical event probabilities				
Cardiovascular death				
Control group	0.00975	0.00877–0.01072	Beta	(9)
Empagliflozin group	0.00864	0.00778–0.00951	Beta	(9)
Hospitalization for heart failure				
Control group	0.02003	0.01803–0.02204	Beta	(17)
Empagliflozin group	0.01466	0.01319–0.01613	Beta	(17)
Readmission for heat failure	0.417	0.3753–0.4587	Beta	(18)
Probability of non-CV mortality by age				
65–69 years	0.2430%			(19)
70–74 years	0.3042%			(19)
75–79 years	0.4185%			(19)
Utility				
NYHA I	0.825	0.790–0.860	Beta	(20)
NYHA II	0.780	0.750–0.810	Beta	(20)
NYHA III	0.650	0.610–0.690	Beta	(20)
NYHA IV	0.585	0.510–0.660	Beta	(20)
Hospitalization and readmission	−0.1	−0.13 to −0.08	Beta	(21)
Cost				
Standard therapy	$131.96	$131.957–310.832	Gammma	(22)
Empagliflozin	$ 59.625	$47.7–71.55	Gammma	Local data
Hospitalization and readmission	$1,783.39	$1029.73–3336.39	Gammma	(23)
Discounted rate	5%	0–8%		(16)

### Simulated population

The clinical characteristics of the simulated population in this model were consistent with the EMPEROR-Preserved trial, including ejection fraction >40%, the same NYHA class I-IV distribution, and the N-terminal pro-brain natriuretic peptide >600 pg/mL (1,200 pg/mL for patients with atrial fibrillation). All cohort populations received standard therapy including diuretics, SAC/VAL, ACEI, ARB, beta-blockers, and spironolactone with or without cardiac implants (9). According to the study of China HF outcome registration, the average age of HFpEF patients in the model was 66 years and the majority of HFpEF were the elderly in the real world (24).

### Clinical event probabilities

An assumption in our model was proposed that the risk of CV death and hospitalization for HF remained unchanged with age because we could not obtain these data from the EMPEROR-Preserved trial (9). The relevant clinical data for the empagliflozin group and control group were derived from the EMPEROR-Preserved trial, the rate of CV death was 7.2% and 8.3%, and the rate of hospitalization for HF was 12.1% and 16.2%, respectively (9, 17). Some data came from other literature instead of EMPEROR-Preserved trial. The rate of readmission for HF within 30 days was 18%, coming from the I-Preservevd trial (18). Age-dependent non-CV deaths were all from the Report on China's Cause of Death 2018, which was published by the China Center for Disease Control and Prevention (19). The formula *r* = −1/t ln(S), *P* = 1-e∧(-r^*^T) was applied to acquire the clinical event probabilities (S is the rate, t is the time, and P is the clinical event probabilities) (25) (Table 1). The transition between different NYHA function classes at the end of every cycle was assumed to be similar between both groups owning to the lack of the effect of empagliflozin on NYHA function classes. The 3-month transition probability between NYHA function classes was derived from published literature (26) (Table 2).

**Table 2:** New York heart association classes transition probabilities per cycle (3 months).

**To**	**I**	**II**	**III**	**IV**	**Distribution**
From					
I	0.977	0.019	0.004	0	Dirichlet
II	0.008	0.981	0.010	0.001	Dirichlet
III	0	0.034	0.960	0.006	Dirichlet
IV	0	0	0.055	0.945	Dirichlet

### Cost and utility

The costs in this model were involved with the cost of hospitalization for HF, standard therapy, and empagliflozin. The cost of hospitalization for HF came from the national statistical database which collected the cost of hospitalization all over the country; therefore, the average cost of hospitalization for HF was $1,783.39 (23). Although HFpEF therapy lacked specific drugs, many were already treated with diuretics, SAC/VAL, ACEI, ARB, beta-blockers, and spironolactone. The cost of standard therapy derived from the national claims sampling database was $131.96 in 2022 at a discount rate of 5% (22). According to the latest national negotiation price in 2022, empagliflozin was $0.6625 per 10 mg, enalapril was $0.088 per 10 mg, SAC/VAL was $0.497 per 100 mg, and the cost of SAC/VAL each cycle was $178.875 (target dose 200 mg, twice daily), so we calculated the range of standard therapy (Table 1). All costs of this study were converted into US dollars at the exchange rate of US $1 = 6.4 yuan (27).

HF was graded based on the ability of daily activities. The utility of every NYHA function class was also different, and the quality-adjusted life years (QALYs) were used as a measurement index. The health utility of patients with HFpEF was derived from the study about indirect, direct non-medical costs and QoL by NYHA classification in Chinese heart failure patients (20). Hospitalization for HF tended to produce a negative effect on healthy life quality and so each hospitalization of HF would reduce the utility value by 0.1 (21) (Table 1).

### Outcome

This model predicted the cardiovascular mortality and the average survival time of the simulated population. The primary endpoint of this study was incremental QALYs and incremental cost, and the incremental cost-effectiveness ratio (ICER) was calculated by the difference in outcomes and the ICER was used to determine the magnitude of the increased costs for each unit in health improvement. According to the recommendation of the World Health Organization (WHO) on the evaluation of Pharmacoeconomics (16): ICER <1-fold of gross domestic product (GDP) per capita, the increased cost is completely worth it and very cost-effective; 1-fold of GDP per capita < ICER <3-fold of GDP per capita, the increased cost is acceptable and cost-effective; and ICER >3-fold of GDP per capita, the increased cost is not worth it and not cost-effective. Since there was no fixed willingness-to-pay (WTP) to evaluate cost-effectiveness in China, we recommended the WTP threshold of $12,652.5 and $37,687.5, which was associated with the one-time and three-times GDP per capita of China in 2021, to determine whether adding empagliflozin in HFpEF was very cost-effective (ICER ≤ $12,652.5) or acceptable (i.e., ICER ≤ $37,687.5) (28).

### Sensitivity analyses

One-way sensitivity analyses were performed to verify the uncertainty of the model by calculating ICER value based on a reasonable range of parameters to make the model more stable according to the recommendations of the Chinese Pharmacoeconomic Evaluation Guide 2019 (16). The 95% confidence intervals of some parameters in the model can be obtained from the published literature. For event probability and medical cost without a specified range, the assuming range was ±10% and ±20%, respectively, and the annual discount rate is in the range of 0–8% (Table 1). The results were presented as a tornado diagram.

Probabilistic sensitivity analysis (PSA) verified the uncertainty of the model in the form of Monte Carlo simulation, which was completed by calculating the random sample results from different input parameter distributions in 1,000 repetitions. The cost parameters adopted gamma distribution, the utility parameters and event probability parameters adopted beta distribution, and the results were represented by cost-effectiveness-acceptability curves (CEACs) and scatter diagram.

Scenario analysis was used to explain that some scenarios had a significant impact on ICER. First, we explored the effect of the national purchase price of empagliflozin ($0.275 per 10 mg, once daily) in 2022 on ICER; Second, the time horizon should be extended to 15 and 20 years to evaluate the uncertainty caused by the time length; Third, we explored the effect of different NYHA function classes on ICER; Fourth, HFpEF with comorbidity or higher level of the hospital had a higher hospitalization: town-level hospitals ($1,029.73), county-level hospitals ($1,231.06), municipal hospitals ($1,783.39), provincial hospitals ($1,949.55), and ministerial hospitals ($3,336.39) (23), so the cost of hospitalization for HF could affect the ICER.

## Results

### Model validation and clinical results

At the end of the EMPEROR-Preserved trial, with a mean follow-up of 27.2 months, the rate of CV death was 7.2% and 8.3% in the empagliflozin group and the control group (9). Our model predicted that the cardiovascular mortality of the empagliflozin group and the control group at 27 months was 8.7% and 9.5%, respectively, and the median survival time of the empagliflozin group and the control group was 16.5 and 14.5 years, respectively, indicating that the clinical outcome predicted by our model was relatively reliable and true.

### Base-case cost-effectiveness analysis

Table 3 represented the total discounted costs and QALYs of the two therapy strategies during the 10 years. Compared with the control group, the add-on empagliflozin treatment increased by 1,271.27 $ and 0.11 QALYs, and the ICER was $11,292.06 per QALY, which was lower than the one-time GDP in 2021 in China. The add-on empagliflozin treatment for HFpEF was a cost-effective option.

**Table 3:** The results from base-case analysis.

	**Total cost ($)**	**Total life years (QALY)**	**Incremental cost ($)**	**Incremental life years (QALY)**	**ICER ($ per QALY)**
Empagliflozin group	5,916.50	4.81	1,271.27	0.11	11,292.06
Control group	4,645.23	4.70			

### Sensitivity analyses

**Figure 2:**
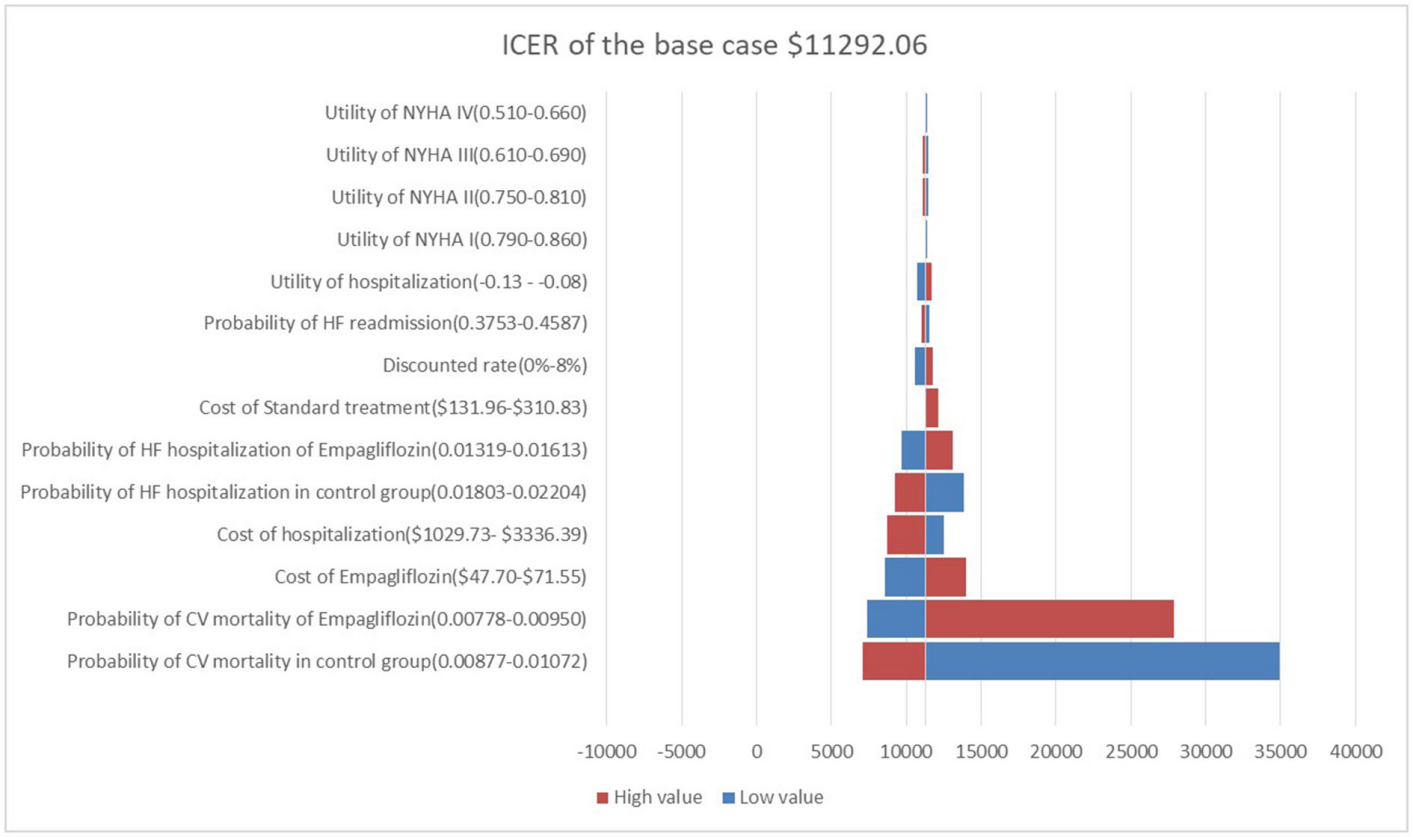
Tornado diagram showing the univariate sensitivity analysis of the Markov model simulation.

One-way sensitivity analysis showed that the ICER calculated by changing a reasonable range of parameters was represented as a tornado diagram (Figure 2), in which the CV death of both groups was the main driver for cost-effectiveness, followed by the cost of empagliflozin and the cost of hospitalization for HF which was higher than one-time GDP but lower than three-times GDP. Changes in the quality of life of stable or worsening HF patients had little effect on ICER.

**Figure 3:**
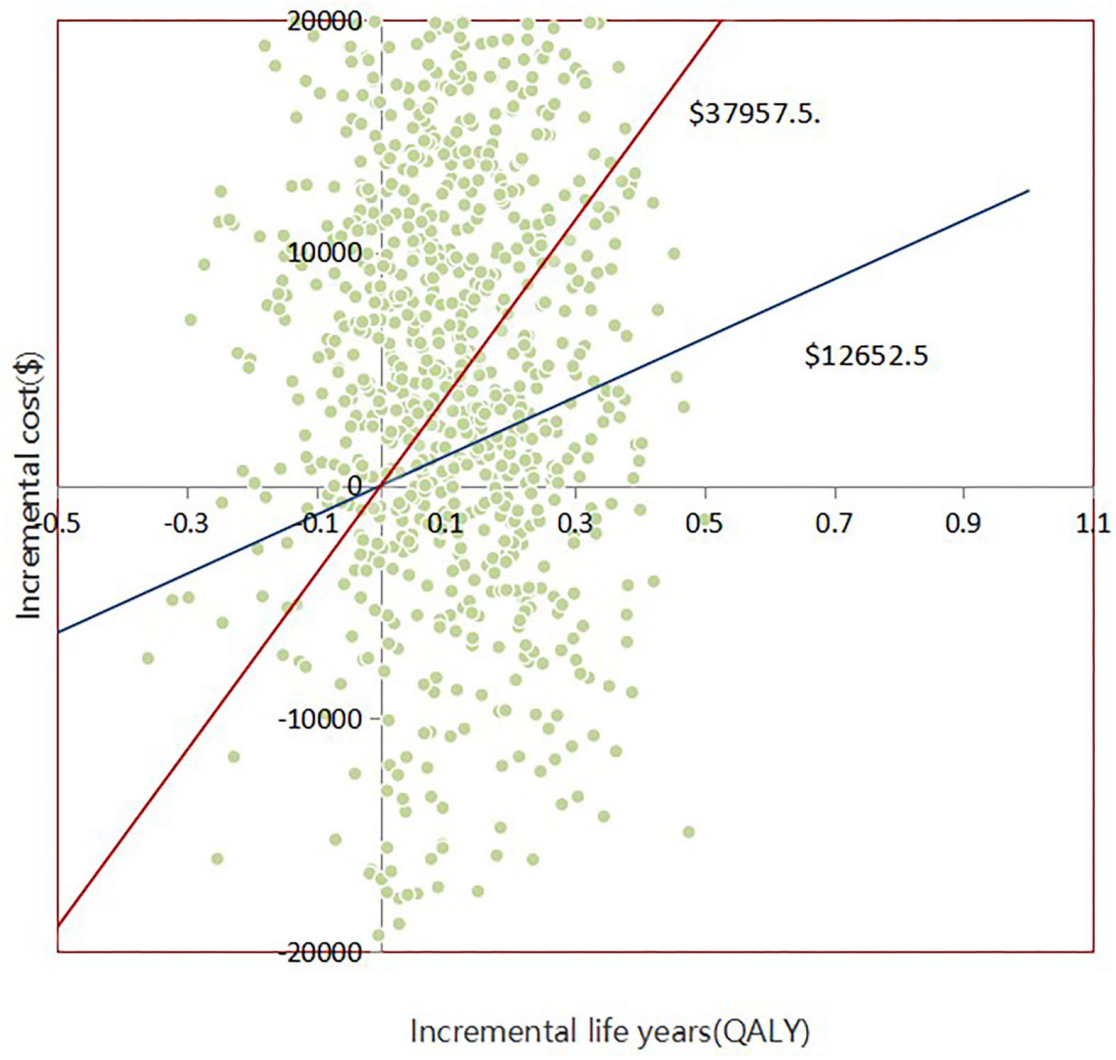
Scatter plot showing the incremental costs and incremental quality-adjusted life-year of a thousand simulations for Empagliflozin group and Control group.

**Figure 4:**
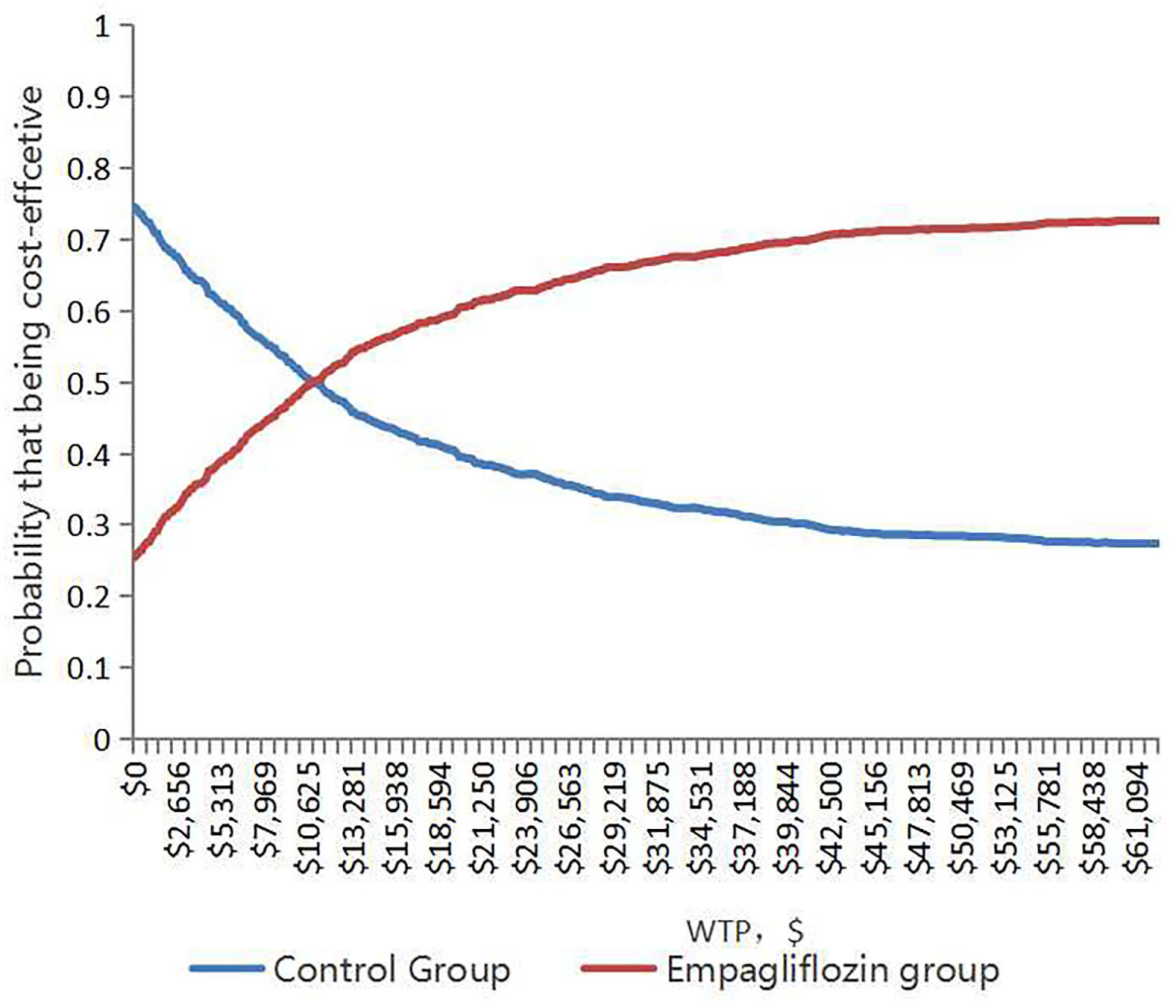
The cost-effectiveness acceptability curve shows the maximum willingness to pay and the corresponding probability of cost-effectiveness for the Empagliflozin group and Control group.

With 1,000 iterations, most ICERs fell in the upper-right quadrant, indicating that the add-on empagliflozin treatment usually produced higher costs and improved QALYs (Figure 3). PSA results revealed that when the WTP threshold was $12,652.5 and $37,957.5, the probability of being cost-effective for using add-on empagliflozin was 52.7 and 67.6%, respectively (Figure 4).

Based on scenario analysis, it was more cost-effective to add empagliflozin treating NYHA I, III, and IV HFpEF patients. The lower national purchase price of empagliflozin, the higher cost of hospitalization for HFpEF with comorbidity, and the longer time horizon contributed to more cost-effectiveness (Table 4).

**Table 4:** The result of scenario analyses presented as ICER.

**Scenario**	**Empagliflozin**
	**ICER ($ per QALY)**
NYHA functional class	
NYHA I	7,243.86
NYHA II	14,044.88
NYHA III	7,891.87
NYHA IV	9,256.53
Price for empagliflozin	
National negotiation price	11,292.06
National purchase price	3,292.60
Hospital level	
Town hospital	12,548.98
County hospital	12,213.21
Municipal hospital	11,292.06
Provincial hospital	11,014.95
Ministerial hospital	8,702.03
Time horizon	
10 years	11,292.06
15 years	9,894.28
20 years	8,995.04

## Discussion

The study explored the cost-effectiveness of adding empagliflozin to standard therapy for patients with HFpEF based on the Markov model. HFpEF patients spent $11,292.06 for each QALY, which was lower than the one-time GDP in China. Adding empagliflozin to standard therapy for patients with HFpEF was cost-effective. One-way sensitivity analysis, PSA, and the scenario analysis were applied to ensure the robustness of the results. The add-on empagliflozin treatment for HFpEF could not only reduce the risk and cost of hospitalization related to HF or outpatient or emergency but also improve the quality of life, mainly because chronic clinical events seriously affected the quality of life (29). Overall, our study provided a valuable quantitative assessment of empagliflozin for medical decision-makers and healthcare payers.

In the sensitivity analysis, it was found that CV death in both groups was the most sensitive to the ICER. Although the risk of CV death was not statistically different between the two groups (hazard ratio, 0.92; 95% CI: 0.75–1.12) (9), comparing outcomes using absolute risk differences provided an alternative picture of therapy benefits, prompting us to explore the cost-effectiveness of adding empagliflozin to standard therapy. If empagliflozin could reduce more CV deaths, the empagliflozin group would produce more QALYs, and the ICER would be lower and more cost-effective.

Data on CV death came from the general population, and the rate of CV death in the Asian population was greater than that in the general population (9), and the ICER was more cost-effective in reality. On the other hand, costs of empagliflozin and hospitalization for HF substantially influenced the pharmacoeconomic benefits, the national purchase price of empagliflozin ($0.275 per 10 mg, once daily) brought more pharmacoeconomic benefits based on the scenario analysis (ICER of $3,292.60 per QALY). The higher the cost of hospitalization, the more cost-effective the adding empagliflozin. For example, the prevalence of HF in China was 1.3%, an increase of 44.0% from 2000 and there are 8.9 million HF patients, and our model predicted that adding empagliflozin to standard therapy could reduce about 17,800 hospitalizations a year and save the cost of hospitalization by $30 million a year. Adding empagliflozin could also reduce the incidence of hospitalizations for HF requiring cardiac care unit/intensive care unit care, emergency or urgent care visits for worsening HF requiring intravenous therapy outpatients, and outpatient intensification of diuretics additionally (17). In the scenario analysis of the time horizon, the longer the time horizon, the more pharmacoeconomic benefits yielded by adding empagliflozin. HF was a chronic disease and required long-term medication. When the simulated horizon was longer than 0.75 and 8 years, respectively, the ICER was lower than the WTP threshold of one-time and three-times GDP. Although NYHA II HFpEF patients spent more cost to gain a QALY due to over 80% of NYHA II HFpEF patients from the EMPEROR-Preserved trial, adding empagliflozin was still cost-effective.

Cost-effectiveness analyses of the add-on empagliflozin in HFrEF had also been proved to be cost-effective in China, the Asia-Pacific Region, and Thailand (30–32), but at the local Thai threshold of 4,773.27 $/QALY, adding empagliflozin to standard therapy yielded a QALY gain of 0.05 at an increased total cost of $622.49 compared to standard therapy alone (ICER: 11,809 $/QALY gained), in which empagliflozin was not a cost-effective add-on treatment for patients with HFpEF (32). We thought the local Thai threshold was much lower than our WTP. Reifsnider et al. reported an economic evaluation of empagliflozin combined with standard therapy for HF patients from healthcare system perspectives in America and the United Kingdom. However, the relevant parameters in that study came from subgroup data from the EMPA-REG OUTCOME trial, which included HFrEF. So the study might not fully reflect the pharmacoeconomic advantages of empagliflozin in HFpEF (33). PSA revealed that when the WTP threshold was $12,652.5 and $37,957.5, the probability of being cost-effective for using add-on empagliflozin was 52.7 and 67.6%, respectively, differing from some published cost-effective study, whose probability was mostly higher than 90% (21, 25), owing to the expensive healthcare expenditure and the higher WTP threshold. Our results were in keeping with basic national conditions and suitable for the healthcare system in China.

To our best knowledge, we are the first to study the pharmacoeconomics of the add-on empagliflozin treatment for HFpEF. Previous studies have focused on HFrEF or HF as a homogeneous group (34). I-Preservevd trial, CHARM-Preserved trial, PARAGON-HF trial, and TOPCAT trial showed that irbesartan, candesartan, SAC/VAL, and spironolactone did not reduce the risk of CV or hospitalization for HF among patients with HFpEF (7, 35, 36). Empagliflozin was the first to be proved to improve HFpEF by a randomized controlled trial with sufficient statistical efficacy. Some molecular mechanisms proposed that empagliflozin could improve the specific circulating microRNA to regulate the endothelial function significantly and empagliflozin could reduce inflammatory and oxidative stress in HFpEF to decrease pathological cardiomyocyte stiffness by inhibiting NHE1 (Na+/H+ exchanger 1), which supported the wide use of empagliflozin in HFpEF (37–39). However, over 86% of HFpEF were using ACE-I/ARBs, 80% were using beta-blockers, and over 24% were using spironolactone in reality (7), which increased the burden of treating HFpEF to some extent. Although there were no head-to-head trials to compare the results of irbesartan, candesartan, SAC/VAL, and spironolactone, because of clinical effects from previous studies or pharmacoeconomic benefits from our cost-effectiveness analysis, empagliflozin was the first choice for HFpEF.

HFpEF patients had many comorbidities including diabetes, half of HFpEF patients in the EMPEROR-Preserved trial had diabetes (9). Although studies have shown that empagliflozin with or without metformin was cost-effective in the treatment of T2DM (40, 41), there was no cost-effective study on the subgroups analysis of HFpEF with diabetes, and clinical outcomes in the diabetes subgroup were similar to that in the general population (9). Adding empagliflozin to standard therapy was a well cost-effective choice for HFpEF with T2DM.

This was a mathematical model combined with national conditions in China, which was suitable for healthcare system in China but may not be suitable for other countries. Hospitalization for non-HF was not considered in our model, but the EMPEROR-Preserved trial showed that empagliflozin could also reduce the risk of all-cause hospitalization by 7% (9). Our results may be more reliable when considering this condition. It was worth mentioning that data on clinical event and utility were derived from other countries, which might cause racial bias to some extent and our study lacked relevant data for subgroup analysis. The fixed parameters such as event probability and utility were also the limiting factors in our model, because these parameters would change with age or interruption of treatment, but the sensitivity analysis explained that our model was robust over a relative wide range of parameters. We assumed that HFpEF in the model could tolerate each drug, ignoring the adverse events but the EMPEROR-Preserved trial showed that the most common adverse events including urinary tract infection, hypovolemia, renal failure, amputation, diabetic ketoacidosis, and gangrene were not significantly different (9). Other possible treatment options in the real world such as drug conversion, drug compliance, or heart transplantation had not been measured. Indirect costs such as productivity loss and disability for HF were not considered in the model because our primary objective was to compare the cost-effectiveness of adding empagliflozin to standard therapy in HFpEF. The model structure in this cost-effectiveness analysis focused on only the CV outcomes of HFpEF patients and other clinically important diseases such as kidney diseases were not considered.

## Conclusion

Generally, from the perspective of the healthcare system in China, this study found that adding empagliflozin to the standard therapy for HFpEF is regarded as a cost-effective option, which provided new insights for medical decision-makers. We believed that our findings would provide some guidance for cost-effective analysis in other countries.

## Data availability statement

The raw data supporting the conclusions of this article will be made available by the authors, without undue reservation.

## Author contributions

YJ collected the data regarding heart failure, analyzed the data, and developed a Markov model. Both authors have read and approved the final manuscript.

## Conflict of interest

The authors declare that the research was conducted in the absence of any commercial or financial relationships that could be construed as a potential conflict of interest.

## Publisher's note

All claims expressed in this article are solely those of the authors and do not necessarily represent those of their affiliated organizations, or those of the publisher, the editors and the reviewers. Any product that may be evaluated in this article, or claim that may be made by its manufacturer, is not guaranteed or endorsed by the publisher.

## Abbreviations

QALY: Quality-Adjusted Life Year
ACEI: Angiotensin-Converting Enzyme Inhibitor
HFrEF: Heart Failure with Reduced Ejection Fraction
HFpEF: Heart Failure with Preserved Ejection Fraction
EMPEROR-Preserved: The Empagliflozin Outcome Trial in Patients with Chronic Heart Failure with Preserved Ejection Fraction
T2DM: Type 2 Diabetes Mellitus
Sac/VAL: Sacubitril/Valsartan
ICER: the Incremental Cost-Effectiveness Ratio
WTP: the Willingness to Pay
ARB: Angiotensin Receptor Antagonists
ARNI: Angiotensin Receptor-Neprilysin Enzyme Inhibitor
SGLT-2 Inhibitor: Sodium-Glucose Cotransporter 2 Inhibitor
WHO: the World Health Organization
GDP: Gross Domestic Product
CEACs: Cost-Effectiveness Acceptability Curves
NYHA: New York Heart Association
CV: Cardiovascular.
