# Corrigendum: Cost-effectiveness of adding empagliflozin to the standard therapy for Heart Failure with Preserved Ejection Fraction from the perspective of healthcare systems in China

**DOI:** 10.3389/fcvm.2023.1205721

**Published:** 2023-05-09

**Authors:** Yaohui Jiang, Jun Xie

**Affiliations:** Department Cardiology, Nanjing Drum Tower Hospital, Affiliated Hospital of Medical School, Nanjing University, Nanjing, China

**Keywords:** empagliflozin, heart failure with preserved ejection fraction, cost-effectiveness analysis, China, pharmaco economic

A Corrigendum on Cost-effectiveness of adding empagliflozin to the standard therapy for Heart Failure with Preserved Ejection Fraction from the perspective of healthcare systems in China by Jiang Y and Xie J. (2022) Front. Cardiovasc. Med. 9:946399. doi: 10.3389/fcvm.2022.946399

In the published article, there was an error in the affiliation. Instead of “Department of Cardiology, Drum Tower Hospital, Nanjing University Medical School, Nanjing, China”, it should be “Department of Cardiology, Nanjing Drum Tower Hospital, Affiliated Hospital of Medical School, Nanjing University, Nanjing, China”.

The authors apologize for this error and state that this does not change the scientific conclusions of the article in any way. The original article has been updated.

